# Life should be redefined

**DOI:** 10.12688/f1000research.151912.1

**Published:** 2024-07-03

**Authors:** Zheng Liu

**Affiliations:** 1 College of Laboratory Medicine, Guilin Medical University, Guilin, China

**Keywords:** DNA replication, reproduction, RNA world, entropic forces, self-replicating molecules

## Abstract

Understanding the nature of life and its propensity for reproduction has long been a question that humans aspire to answer. Reproduction, a defining characteristic of life, fundamentally involves the replication of genetic material, be it DNA or RNA. The driving force behind this replication process has always intrigued scientists. In recent years, theories involving selfish genes, the RNA world, and entropic forces have been proposed by some scholars. These theories seem to suggest that life, as we know it, exists solely in Earth’s environment and is based on a single type of genetic material, either DNA or RNA. However, if we broaden our definition of life to include any replicable molecules, we might be able to transcend traditional thought. This could potentially enhance our understanding of the impetus behind DNA replication and provide deeper insights into the essence of life.

## Introduction

The quest for understanding the existence of life has always captivated people. Currently, it’s challenging to define what life is. Traditionally, Life is defined as an extension of being into the next generation and into the next species in evolutionary time ( Chatterjee, 2023). Reproduction, the process of passing on genetic material (DNA or RNA) to the next generation, is a fundamental characteristic of life. This phenomenon prompts the question: why is the replication of DNA or RNA necessary for life and what drive DNA or RNA replication? Over the years, scientists have deepened their understanding of life. Some have proposed the theory of selfish genes ( Noble and Noble, 2022), while others argue that RNA is the origin of life ( Higgs and Lehman, 2015). A particularly innovative perspective comes from Professor Jeremy England, who posits that entropic forces drive the replication of DNA or RNA ( England, 2013). His theory, grounded in the second law of physics, suggests that life arises from the increase in the universe’s entropy. This provides a physical explanation for the reproduction and continuation of life. Building on these theoretical foundations, I think the life and genetic materials should be redefined. Given that DNA or RNA, as genetic material, can replicate and generate life, I hypothesize that any molecules with replication capabilities, regardless of their environment, could potentially evolve into life. This speculation opens up possibilities for the discovery of new life forms in the universe.

## The expectation for reproduction of organisms

Why is there such a vast diversity of life on Earth? The prevailing understanding is that given varying environmental pressures and ample time, a single species can evolve into distinct species. Each species has a natural desire to reproduce and pass on its genes. As Charles Darwin elucidated, individuals possessing traits that better suit their environment have a higher likelihood of reproducing. This allows them to pass on these advantageous traits to their offspring, thereby enhancing the organism’s reproductive success and allowing the organism to reproduce more. Over time, this process results in the evolution of species that are well-adapted to their specific environments. The innate drive to survive and reproduce is fundamental to the perpetuation and diversity of life on Earth. But what exactly is the purpose behind the reproduction of species? Humans have established complex societies, systems of relationships and norms, to fulfil their social, emotional, and cultural needs. Naturally, human reproduction encompasses these needs, including sexual maturation, social support, and the continuation of the family line. In today’s world, there is no doubt that social and emotional needs have become paramount. However, these needs are not present in viruses, bacteria, or insects. Although viruses and bacteria seem to lack self-consciousness, they still carry out self-replication. A fascinating example is the male mayfly, which has a lifespan of only 24 hours ( Araújo et al., 2021). Its sole purpose from birth is to mate. What is the objective of this replication in bacteria and viruses and what drives male mayflies to behave in such a way?

## Selfish genes drive reproduction

**Figure 1.**
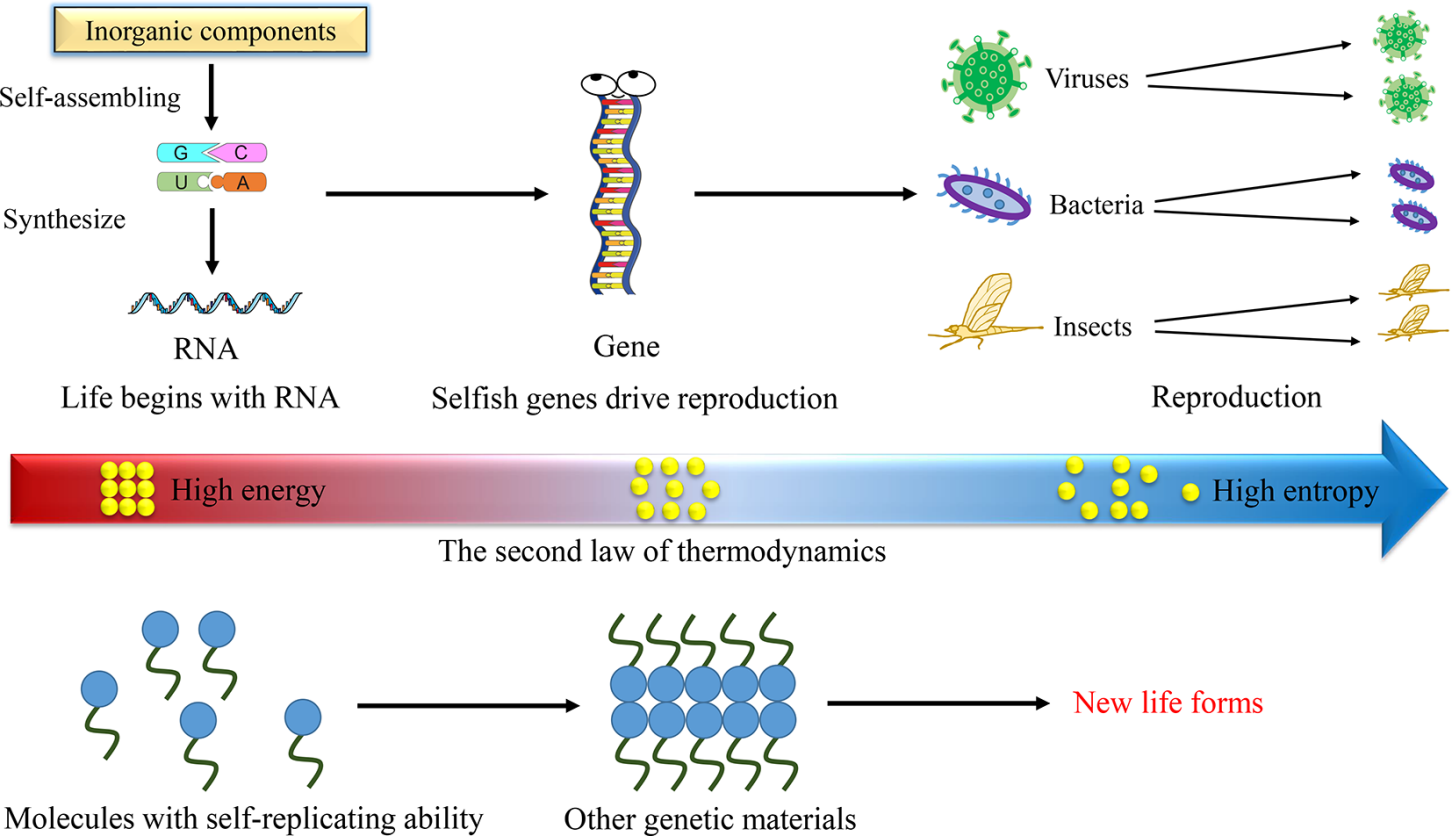
Entropic forces drive the evolution, leading to the generation of new life forms. On Earth, inorganic molecules capable of self-replication spontaneously synthesize organic molecules, including RNA. Influenced by the entropic forces, RNA serves as the genetic material, leading to the emergence of life, irrespective of whether the organisms possess self-consciousness. In environments distinct from Earth, any molecules with self-replication capabilities could potentially evolve into genetic material, thereby initiating new forms of life.

One theory suggests that genes choose us, and they are selfish ( Gardner and Welch, 2011). In his book, ‘The Selfish Gene’, Richard Dawkins posits that genes, rather than individuals or species, are the primary units of selection in evolution. Consequently, genes have propelled the reproduction of all species. According to this theory, genes are the fundamental unit of natural selection, acting in a manner that ensures their own survival. Organisms serve as carriers of these genes, with reproduction being the ultimate goal ( Figure 1). Reproduction is the prize for living organism with or without self-consciousness. Numerous instances of this theory are evident in nature, and recent studies have further validated it. A study found that individuals with specific variants of the ERAP2 gene had a higher survival rate during the Black Death, a catastrophic pandemic in the 14 ^th^ century ( Klunk et al., 2022). This pandemic, from another perspective, is that ERAP2 gene with specific variants, selectively replicated itself within certain individuals. Such events have recurred throughout human history, exerting substantial selective pressure on the human population. This altered the prevalence of certain genetic variants, thereby influencing our disease susceptibility today. From this perspective, it appears as though the ERAP2 gene with specific variants is driven by its survival instinct. However, this theory does not address the question of why genes, despite their lack of self-consciousness, need to replicate and ensure their survival. In other words, what is the purpose of gene replication?

## Life begins with RNA

In order to address the aforementioned questions, it is essential to determine what the first gene or genetic material on Earth was and understand how it initiated replication. The RNA world hypothesis suggests that life on Earth have begun with a simple self-replicating RNA molecule, with DNA only appearing later as a result of RNA evolution. Gerald Joyce team discovered that a highly evolved RNA polymerase ribozyme has the ability to function as a reverse transcriptase ( Samanta and Joyce, 2017). This reverse transcriptase ribozyme can incorporate all four types of dNTPs and can produce products containing up to 32 deoxynucleotides. Recently, significant progress has been made by the team led by Clemens Richert. They managed to replicate RNA sequences of up to 12 nucleotides in a dilute aqueous solution of an unactivated dinucleotide, without the involvement of any enzymes ( Welsch et al., 2023). The RNA world hypothesis offers a persuasive explanation for the early stages of life on Earth. However, is RNA an organic molecule that can be directly synthesized from inorganic substances? The Miller-Urey experiment in 1953 was provided evidence that essential organic molecules, fundamental to life, could be synthesized from inorganic substances. In fact, scientists have developed more methods in recent years to synthesize organic molecules from hydrogen and oxygen under different conditions ( Hudson et al., 2020). Studies have demonstrated that all four of RNA’s major components can be efficiently assembled from simple chemicals under certain conditions ( Figure 1). A paper published in Science presents a straightforward chemical pathway to the formation of ribonucleotides ( Becker et al., 2016). These studies underscore the remarkable ability of the natural environment to transform simple inorganic molecules into complex organic ones, such as RNA, a key characteristic of life on Earth. It suggests that the emergence of life could be a random event in nature, rather than being designed. However, what drives RNA to replicate itself?

## Entropic forces drive life

Jeremy England, a professor at Massachusetts Institute of Technology, has put forth a theory suggesting that life arises because the principle of increasing entropy compels matter to adopt life-like physical properties ( England, 2015). It would suggest that the replication of genes is driven by entropic forces ( Figure 1). This theory has been employed to elucidate the impetus behind evolution and the replication of DNA/RNA. DNA/RNA molecules prefer to be in their lowest energy conformation (the resting position with high entropy). Replication of DNA or RNA is a process that facilitates the attainment of this state. Interestingly, this theory is a manifestation of the renowned Big Chill hypothesis (or the heat death of the universe) in the context of human evolution. The Big Chill hypothesis posits that all entities in the universe are transitioning from a state of uniform high energy to one of high entropy based on the second law of thermodynamics ( Fleck, 2023). Therefore, it can be inferred that every living organism will attain a state of high entropy. Molecules with high entropy are both disordered and disorganized. A study discovered that an increase in entropy leads to a more random replication process, potentially resulting in a higher rate of errors or mutations ( Arias-Gonzalez., 2012). Actually, gene mutations are essential for the survival of genes under stress. If a mutation provides an organism with an environmental advantage, that organism has a higher likelihood of survival and reproduction. This phenomenon is evident in the continuous mutation of the SARS-CoV-2 virus as it attempts to coexist with humans ( Markov et al., 2023).

## RNA and DNA are not the only genetic materials

If all the theories mentioned above are proven correct, it leads us to several intriguing questions. Why is life, as we know it, only discovered on Earth so far? To date, there are no known types of genetic material fundamentally different from DNA and RNA. Since anywhere in the universe will transit from a state of low entropy to high entropy, does this imply that only Earth’s environment is suitable for DNA/RNA replication? In other words, does life in the universe require an environment similar to Earth’s to exist? If we assume that the only genetic material for life is DNA or RNA, then it implies that there is only one form of life in the universe, akin to life on Earth. It suggests that only another planet with similar conditions - air, water, temperature, pressure, and so on - could give rise to DNA or RNA, and subsequently, organisms resembling those on Earth. Do we increasingly believe that there is only life on Earth in the universe, or that only environments similar to Earth can produce life? Moreover, life has only one form, which is a living organism with DNA or RNA as genetic material. I disagree with this viewpoint. This perspective is inherently flawed as it habitually considers DNA and RNA as the sole genetic materials. The crux of the matter is that DNA or RNA may be the only genetic materials suitable for Earth’s environment.

## Any molecules with self-replicating ability can be genetic material

Perhaps a shift in our perspective could provide a better answer to this question. We could redefine the genetic material of life (or living organisms) as any molecules capable of self-replication, not limited to DNA or RNA ( Figure 1). In this way, life needs to be redefined. Actually, self-replicate has been seen in very different looking chemical systems, for example in rotaxanes, a self-assembling rotaxane that can replicate itself discovered by the scientists ( Tamara et al., 2015). Ignacio Colomer et al. discovered a self-replicator consists of a system of small molecules composed of hydrogen and carbon ( Colomer et al., 2018). This process of self-replication could occur anywhere in the universe, which encompasses a vast range of environments distinct from Earth. For instance, envision this process taking place in an atmosphere with a temperature of -63 degrees Celsius, a pressure of 600 Pa, and a composition of 95% carbon dioxide - conditions naturally found on Mars. Currently, the laboratory possesses the capability to simulate the self-replication of substances under such conditions. Actually, Schulze-Makuch et al., has already proposed alien life could be based on different chemistries, solvents, and energy sources ( Schulze-Makuch and Irwin, 2006). Given sufficient time, these simple organic molecules, capable of self-replication, could give rise to other forms of life, similar to the original genetic material RNA on Earth. In other words, the genetic material of life elsewhere in the universe may not necessarily be DNA or RNA, as these replicable molecules could potentially form other new life forms.

## Conclusions

This paper presents a novel opinion by reviewing recent studies and theories concerning the biological reproduction, drawing from the fields of biology (with a focus on theories of selfish genes and the RNA world), physics (specifically the second law of thermodynamics and entropy theory), and chemistry (emphasizing self-replicating systems). It suggests that molecules capable of self-replication may, under certain conditions, evolve into various forms of life. This perspective represents a departure from traditional concepts that identify DNA and RNA as the sole genetic materials. It provides a new insight into the origins and implications of life’s existence in the universe, providing a theoretical basis for searching for other diverse forms of life in the universe.

### Ethical approval and consent

Ethical approval and consent were not required.

## Data Availability

No data are associated with this article.
