# Life should be redefined: Any molecule with the ability to self-replicate should be considered life

**DOI:** 10.12688/f1000research.151912.2

**Published:** 2024-09-20

**Authors:** Zheng Liu

**Affiliations:** 1 College of Laboratory Medicine, Guilin Medical University, Guilin, China

**Keywords:** DNA replication, reproduction, RNA world, entropic forces, self-replicating molecules

## Abstract

Understanding the nature of life and its propensity for reproduction has long been a question that humans aspire to answer. Reproduction, a defining characteristic of life, fundamentally involves the replication of genetic material, be it DNA or RNA. The driving force behind this replication process has always intrigued scientists. In recent years, theories involving selfish genes, the RNA world, and entropic forces have been proposed by some scholars. These theories seem to suggest that life, as we know it, exists solely in Earth’s environment and is based on a single type of genetic material, either DNA or RNA. However, if we broaden our definition of life to include any replicable molecules, we might be able to transcend traditional thought. This could potentially enhance our understanding of the impetus behind DNA replication and provide deeper insights into the essence of life.

## Introduction

The quest for understanding the existence of life has always captivated people. Currently, it’s challenging to define what life is. Traditionally, life is defined as an extension of being into the next generation and into the next species in evolutionary time ( Chatterjee, 2023). Reproduction, the process of passing on genetic material (DNA or RNA) to the next generation, is a fundamental characteristic of life. This phenomenon prompts the question: why is the replication of DNA or RNA necessary for life and what drive DNA or RNA replication? Over the years, scientists have deepened their understanding of life. Some have proposed the theory of selfish genes ( Noble and Noble, 2022), while others argue that RNA is the origin of life ( Higgs and Lehman, 2015). A particularly innovative perspective comes from Professor Jeremy England, who posits that entropic forces drive the replication of DNA or RNA ( England, 2013). His theory, grounded in the second law of thermodynamics, suggests that life arises from the increase in the universe’s entropy. This perspective provides a physical explanation for the reproduction and continuation of life. Building on these theoretical foundations, I think the life and genetic materials should be redefined. Given that DNA or RNA, as genetic material, can replicate and generate life, I hypothesize that any molecules with replication capabilities, regardless of their environment, whether organic or inorganic, could potentially evolve into life. This speculation opens up possibilities for the discovery of new life forms in the universe.

## The expectation for reproduction of organisms

Why is there such a vast diversity of life on Earth? The prevailing understanding is that given varying environmental pressures and ample time, a single species can evolve into distinct species. Each species has a natural desire to reproduce and pass on its genes. As Charles Darwin elucidated, individuals possessing traits that better suit their environment have a higher likelihood of reproducing. This allows them to pass on these advantageous traits to their offspring, thereby enhancing the organism’s reproductive success and allowing the organism to reproduce more. Over time, this process results in the evolution of species that are well-adapted to their specific environments. The innate drive to survive and reproduce is fundamental to the perpetuation and diversity of life on Earth. But what exactly is the purpose behind the reproduction of species? Humans have established complex societies, systems of relationships and norms, to fulfil their social, emotional, and cultural needs. Naturally, human reproduction encompasses these needs, including sexual maturation, social support, and the continuation of the family line. In today’s world, there is no doubt that social and emotional needs have become paramount. However, these needs are not present in viruses, bacteria, or insects. Although viruses and bacteria seem to lack self-consciousness, they still carry out self-replication. A fascinating example is the male mayfly, which has a lifespan of only 24 hours ( Araújo et al., 2021). Its sole purpose from birth is to mate. What is the objective of this replication in bacteria and viruses and what drives male mayflies to behave in such a way?

## Selfish genes drive reproduction

**Figure 1.**
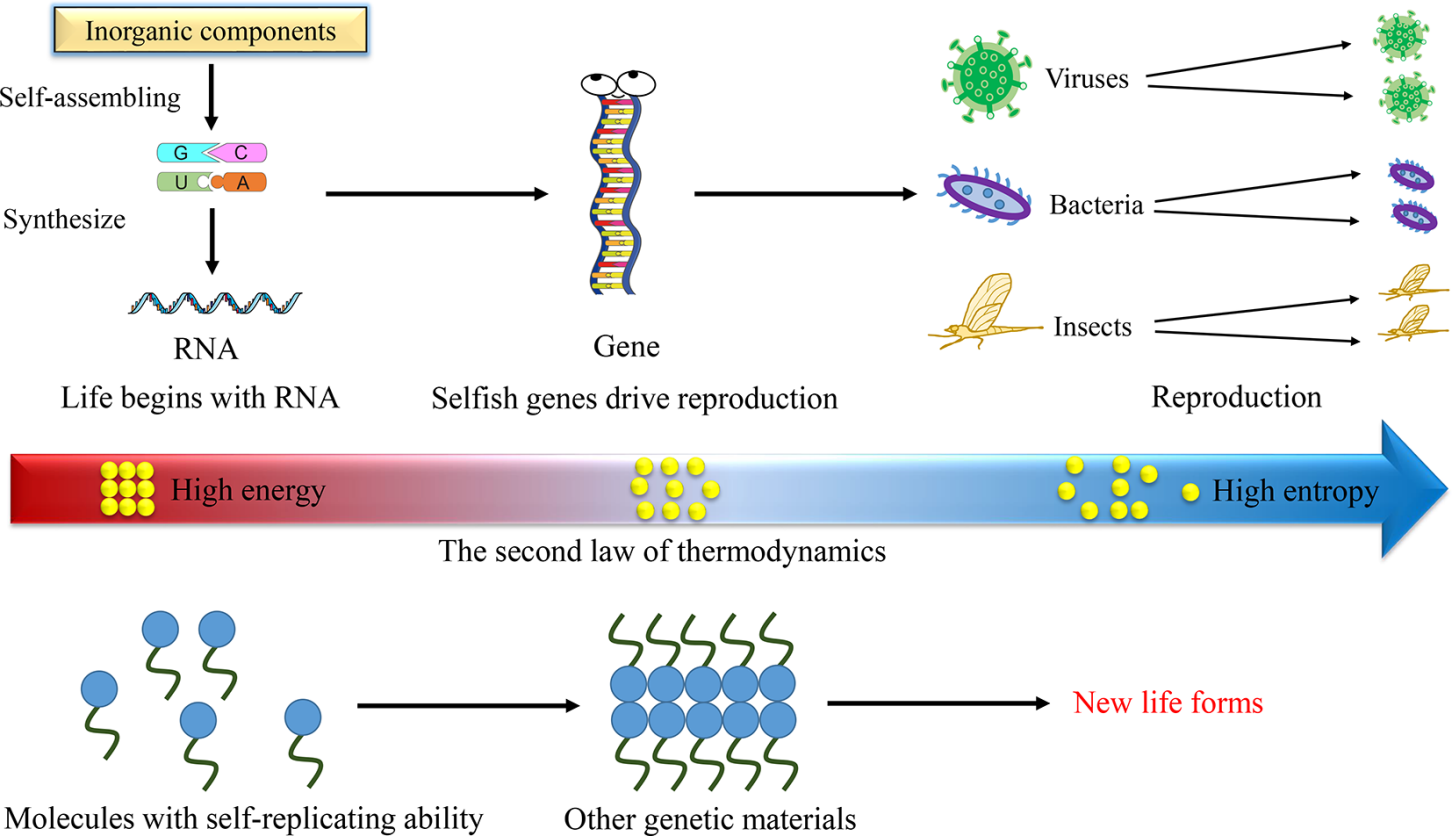
Entropic forces drive the evolution, leading to the generation of new life forms. On Earth, inorganic molecules capable of self-replication spontaneously synthesize organic molecules, including RNA. Influenced by the entropic forces, RNA serves as the genetic material, leading to the emergence of life, irrespective of whether the organisms possess self-consciousness. In environments distinct from Earth, any molecules with self-replication capabilities could potentially evolve into genetic material, thereby initiating new forms of life.

One theory suggests that genes choose us, and they are selfish ( Gardner and Welch, 2011). In his book, ‘The Selfish Gene’, Richard Dawkins posits that genes, rather than individuals or species, are the primary units of selection in evolution. Consequently, genes have propelled the reproduction of all species. According to this theory, genes are the fundamental unit of natural selection, acting in a manner that ensures their own survival. Organisms serve as carriers of these genes, with reproduction being the ultimate goal ( Figure 1). Reproduction is the prize for living organism with or without self-consciousness. Numerous instances of this theory are evident in nature, and recent studies have further validated it. A study found that individuals with specific variants of the ERAP2 gene had a higher survival rate during the Black Death, a catastrophic pandemic in the 14 ^th^ century ( Klunk et al., 2022). This pandemic, from another perspective, is that ERAP2 gene with specific variants, selectively replicated itself within certain individuals. Such events have recurred throughout human history, exerting substantial selective pressure on the human population. This altered the prevalence of certain genetic variants, thereby influencing our disease susceptibility today. From this perspective, it appears as though the ERAP2 gene with specific variants is driven by its survival instinct. However, this theory does not address the question of why genes, despite their lack of self-consciousness, need to replicate and ensure their survival. In other words, what is the purpose of gene replication?

## Life begins with RNA

In order to address the aforementioned questions, it is essential to determine what the first gene or genetic material on Earth was and understand how it initiated replication. The RNA world hypothesis suggests that life on Earth have begun with a simple self-replicating RNA molecule, with DNA only appearing later as a result of RNA evolution. Gerald Joyce team discovered that a highly evolved RNA polymerase ribozyme has the ability to function as a reverse transcriptase ( Samanta and Joyce, 2017). This reverse transcriptase ribozyme can incorporate all four types of dNTPs and can produce products containing up to 32 deoxynucleotides. Recently, significant progress has been made by the team led by Clemens Richert. They managed to replicate RNA sequences of up to 12 nucleotides in a dilute aqueous solution of an unactivated dinucleotide, without the involvement of any enzymes ( Welsch et al., 2023). The RNA world hypothesis offers a persuasive explanation for the early stages of life on Earth. However, is RNA an organic molecule that can be directly synthesized from inorganic substances? The Miller-Urey experiment in 1953 was firstly provided evidence that essential organic molecules, fundamental to life, could be synthesized from inorganic substances. In fact, scientists have developed more methods in recent years to synthesize organic molecules from hydrogen and oxygen under different conditions ( Hudson et al., 2020). Studies have demonstrated that all four of RNA’s major components can be efficiently assembled from simple chemicals under certain conditions ( Figure 1). A paper published in Science presents a straightforward chemical pathway to the formation of ribonucleotides ( Becker et al., 2016). These studies underscore the remarkable ability of the natural environment to transform simple inorganic molecules into complex organic ones, such as RNA, a key characteristic of life on Earth. It suggests that the emergence of life could be a random event in nature, rather than being designed. However, what drives RNA to replicate itself?

## Entropic forces drive life

From a physics perspective, the fundamental difference between living organisms and inanimate clumps of carbon atoms is that living organisms are much more efficient at capturing energy from their environment. This efficiency is largely due to complex biochemical processes, such as photosynthesis in plants and cellular respiration in animals, which allow them to convert energy from their environment into usable forms and dissipate excess energy as heat. Jeremy England, a professor at the Massachusetts Institute of Technology, has derived a mathematical formula to explain this ability. According to this formula, based on established physics, when a group of atoms is driven by an external energy source and surrounded by a heat bath, it will often gradually restructure itself to dissipate increasing amounts of energy ( England, 2015). It would suggest that the replication of genes is driven by entropic forces ( Figure 1). This theory has been employed to elucidate the impetus behind evolution and the replication of DNA/RNA. DNA/RNA molecules prefer to be in their lowest energy conformation (the resting position with high entropy). Replication of DNA or RNA is a process that facilitates the attainment of this state. Interestingly, this theory is a manifestation of the renowned Big Chill hypothesis (or the heat death of the universe) in the context of human evolution. The Big Chill hypothesis posits that all entities in the universe are transitioning from a state of uniform high energy to one of high entropy based on the second law of thermodynamics ( Fleck, 2023). Therefore, it can be inferred that every living organism will attain a state of high entropy. Molecules with high entropy are both disordered and disorganized. An interesting question is, is the theory of entropic forces contradictory to Darwin’s theory of evolution?

## Entropic forces theory as a supplement to Darwin’s theory of evolution

Based on the aforementioned theories and speculations, I propose that the emergence of life is associated with the heat death of the universe and is likely a byproduct of this phenomenon. During the heat death of the universe, the emergence of life was an accidental phenomenon accompanied by a decrease in energy. Importantly, it should be noted that the Entropic Forces Drive Life theory does not contradict Darwin’s theory of evolution; rather, they complement each other. Entropic forces are the driving force behind the emergence of life, explaining why life arises and its ultimate goal (the heat death of the universe). Darwin’s theory of evolution explains how species evolve over time through natural selection, where advantageous traits become more common in a population. It describes the evolution of species adapting to their environment after the emergence of life. Some argue that the increase in complexity (more orderly and organized) seen in evolution contradicts the second law of thermodynamics, which states that systems tend to become more disordered over time ( Vanchurin et al., 2022). A study points out that the second law of thermodynamics applies to closed systems, while living organisms are open systems that exchange energy and matter with their surroundings ( Schreiber and Gimbel, 2010). This allows for local decreases in entropy (increased order) at the expense of increased entropy in the environment. I prefer to compare the Earth’s environment to a small-open system, relative to the universe, which we can understand as a large closed system, similar to the difference between the environment of a house and the entire Earth. This small-open environment may not necessarily follow the second law of thermodynamics at certain times and places. My explanation is that after life emerges, in the face of a small-open environment where entropy may dynamically increase or decrease, life constantly adjusts energy changes to form a steady state with the environment. Therefore, the evolution of life in such an environment can lead to either increased or decreased entropy, all in order to adapt. The new question is whether the Earth is developing towards low entropy or high entropy. Currently, there are no research reports on this. However, most scientists believe that the Earth, like all systems in the universe, is developing towards higher entropy, although the Earth absorbs energy from the Sun. While living organisms on Earth maintain a state of low entropy by consuming energy and creating order within their systems, the energy transformations involved in these processes result in an overall increase in entropy in the universe. Therefore, despite local decreases in entropy, the Earth as a whole is moving towards higher entropy. So, what impact does this small-open environment (on Earth) have on biological evolution?

## The small-open environments hinder the evolution of life

An interesting phenomenon is that when a large number of cells (a large number of genes) form a complex living organism, like humans, they may be better able to adapt to the special small-open environments (on Earth) under selection pressure and obtain energy, forming a steady state with the environment and gaining some survival advantages. However, I believe they also lose the ability to change because it violates the principle of entropic forces in the universe. Like flowers in a greenhouse, when the environment changes, they cannot adapt in time and will face faster death. For example, although humans have evolved to the top of the food chain and can alter some environments through scientific technology (such as by using air conditioning), allowing them to reproduce in large numbers and dominate Earth’s resources and energy, they cannot regenerate a broken tail like geckos, nor do they possess the rapid mutation and adaptation abilities of viruses in response to sudden environmental changes. Unlike humans, bacteria and viruses have not evolved to optimize their energy extraction from the Earth. Consequently, most animals are currently at a disadvantage within the Earth’s ecological system. However, a study discovered that an increase in entropy (decreased energy) leads to a more random replication process, potentially resulting in a higher rate of errors or mutations ( Arias-Gonzalez, 2012). Actually, gene mutations are essential for the survival of genes under stress. If a mutation provides an organism with an environmental advantage, that organism has a higher likelihood of survival and reproduction. This phenomenon is evident in the continuous mutation of the SARS-CoV-2 virus as it attempts to coexist with humans ( Markov et al., 2023). The latest story is about a bacterium that can surprisingly engulf plastic through a novel mutation, a pollutant that humans currently cannot solve ( Kim et al., 2024). I believe the reason is that, driven by entropic forces, some living organisms have strengthened their survival in small environments (e.g., the environment on Earth) while losing their ability to cope with changes in the larger environment (e.g., interstellar or cosmic space). The small-open environment sometimes contradicts the second law of thermodynamics of the larger environment, moving from low energy to high energy. For example, in winter, when a house is heated by air conditioning, people who stay in the room for a long time mistakenly believe that the world has always been warm, and their genes change in response to this warmth. When the house is broken, and the cold air from outside blows in, the creatures in the room are easily frozen to death. Therefore, the entropic forces in small-open environments can only produce fragile organisms, whether or not it is consistent with the entropy forces of the universe, and their ultimate fate is consistent with the entropy energy in the universe (the heat death). This may seem pessimistic, but as described in the science fiction novel “ *The Last Question*,” even if humanity expands to inhabit the entire universe, its ultimate fate will mirror that of the universe itself, moving towards heat death. This raises a new question: Can small-open environments only produce fragile life forms like those on Earth?

## RNA and DNA are not the only genetic materials

If all the theories mentioned above are proven correct, it leads us to several intriguing questions. How many small-open environments exist in the universe? Are they all the same? Why is life, as we know it, only discovered on Earth so far? To date, there are no known types of genetic material fundamentally different from DNA and RNA. Since anywhere in the universe will transit from a state of low entropy to high entropy, does this imply that only Earth’s environment is suitable for DNA/RNA replication? In other words, does life in the universe require an environment similar to Earth’s to exist? If we assume that the only genetic material for life is DNA or RNA, then it implies that there is only one form of life in the universe, akin to life on Earth. It suggests that only another planet with similar conditions - air, water, temperature, pressure, and so on - could give rise to DNA or RNA, and subsequently, organisms resembling those on Earth. All small-open environments in the universe are same. Do we increasingly believe that there is only life on Earth in the universe, or that only environments similar to Earth can produce life? Moreover, life has only one form, which is a living organism with DNA or RNA as genetic material. I disagree with this viewpoint. This perspective is inherently flawed as it habitually considers DNA and RNA as the sole genetic materials. The crux of the matter is that DNA or RNA may be the only genetic materials suitable for Earth’s environment. However, small-open environments across the universe vary significantly. So, what might genetic material and life look like in these different small-open environments?

## Any molecules with self-replicating ability can be genetic material

Perhaps a shift in our perspective could provide a better answer to this question. We could redefine the genetic material of life (or living organisms) as any molecules capable of self-replication, not limited to DNA or RNA ( Figure 1). In this way, life needs to be redefined. Any molecules, whether organic or inorganic, capable of proactive self-replication should be considered life. Importantly, this self-replication is an active process of molecules, rather than the accumulation of products. An interesting example is prions. The prion protein is encoded by the PRNP gene and cannot self-replicate, therefore it cannot be considered life ( Bernardi and Bruni, 2019). However, when the prion protein misfolds, the misfolded prion acts as a template, causing normal proteins to adopt the abnormal structure ( Kupfer et al., 2009). It is much like a zombie infection in the movie *Resident Evil: Welcome to Raccoon City*. Infected individuals transmit the infection to healthy individuals through a specific pathway. This misfolded prion gains the ability of self-replication and possesses the characteristics of life. I think perhaps misfolded prions are in a high-energy and low-entropy state due to changes in protein structure, and can only or optimally reach a high-entropy state by altering the structure of other prions. Actually, self-replicate has been seen in very different looking chemical systems, for example in rotaxanes, a self-assembling rotaxane that can replicate itself discovered by the scientists ( Kosikova et al., 2015). Ignacio Colomer et al. discovered a self-replicator consists of a system of small molecules composed of hydrogen and carbon ( Colomer et al., 2018). This process of self-replication could occur anywhere in the universe, which encompasses a vast range of environments distinct from Earth. For instance, envision this process taking place in an atmosphere with a temperature of -63 degrees Celsius, a pressure of 600 Pa, and a composition of 95% carbon dioxide - conditions naturally found on Mars. Currently, the laboratory possesses the capability to simulate the self-replication of substances under such conditions. Actually, Schulze-Makuch et al., has already proposed alien life could be based on different chemistries, solvents, and energy sources ( Schulze-Makuch and Irwin, 2006). Given sufficient time, these simple organic molecules, capable of self-replication, could give rise to other forms of life, similar to the original genetic material RNA on Earth. In other words, the genetic material of life elsewhere in the universe may not necessarily be DNA or RNA, as these replicable molecules could potentially form other new life forms. An interesting question is, are organisms composed of other molecules with self-replicating abilities more robust? We can imagine life forms composed of different unique substances existing in various small, open environments throughout the universe. Some life forms might adapt to the universe’s environment without the need for air or water. Their bodies could be extremely hard, similar to the droplets in the novel *The Three-Body Problem*, tightly connected by atomic nuclei. Can we further explore the concept of life more boldly at this point in the paper?

## A bold perspective

Science fiction movies engage in open-ended imagination based on scientific knowledge and present it to the audience for viewing. Famous science fiction movies related to the definition of life include “ *Transformers*” and “ *The Thirteenth Floor*”. “ *Transformers*” defines extraterrestrial creatures as steel-like life forms, unlike humans who have soft and fragile bodies. This is an imagination of silicon-based life or other replicable molecular-based life forms. I believe that when more suitable life forms emerge for the harsh environment in the universe, they will replace the more fragile replicable molecular life forms, just as there are prophecies that silicon-based life forms will replace carbon-based life forms (Schulze-Makuch and Irwin 2006). “ *The Thirteenth Floor*” offers another definition of life, leaving the material world, that is, whether the code that can be copied in a computer is considered life. Although this is not within the scope of this article, it is based on the molecules that exist in the material world. However, I don’t think this is contradictory either. Perhaps the material world and the virtual world exist in parallel, with mutual connections and interactions. Our actions in the physical world can create ripple effects in the digital realm, and vice versa. In addition, can radio waves and other rays that are invisible to the naked eye create life? Is Musk’s proposal to upload and store brain information (mind uploading) also considered life? Is the self-aware intelligent robot in movie *I, Robot* considered as life? Is the cyborg combination of humans and machines in the movie *Alita: Battle Angel* considered life? These questions may seem bizarre now, but in the near future, they will be questions that we humans must answer.

## Conclusions

This paper presents a novel opinion by reviewing recent studies and theories concerning the biological reproduction, drawing from the fields of biology (with a focus on theories of selfish genes and the RNA world), physics (specifically the second law of thermodynamics and entropy theory), and chemistry (emphasizing self-replicating systems). It suggests that any molecules, whether organic or inorganic, capable of self-replication may, under certain conditions, evolve into various forms of life. This perspective represents a departure from traditional concepts that identify DNA and RNA as the sole genetic materials. It provides a new insight into the origins and implications of life’s existence in the universe, providing a theoretical basis for searching for other diverse forms of life in the universe. Finally, conclude with three philosophical questions: “Who am I?”, “Where do I come from?”, and “Where am I going”?

### Ethical approval and consent

Ethical approval and consent were not required.

## Data Availability

No data are associated with this article.
