# Supplementary material for: Life should be redefined: Any molecule with the ability to self-replicate should be considered life
Source: F1000Res. 2024 Sep 20;13:736. Originally published 2024 Jul 3. [Version 2] doi: 10.12688/f1000research.151912.2 (PMC11467646; doi:10.12688/f1000research.151912.2)
Supplement: Peer review history

# Reviewer report: Reviewer response for version 2

**DOI:** 10.5256/f1000research.171902.r325497

**Published:** 2024-10-10

**Authors:** Kevin S. Lang

**Affiliations:** 1 University of Minnesota, Saint Paul, MN, USA

I think the author addressed my concerns adequately with their revisions.

Is the topic of the opinion article discussed accurately in the context of the current literature?

Partly

Are arguments sufficiently supported by evidence from the published literature?

Partly

Are all factual statements correct and adequately supported by citations?

Partly

Are the conclusions drawn balanced and justified on the basis of the presented arguments?

Partly

Reviewer Expertise:

DNA Replication, transcription, translation, microbiology, molecular biology, evolution.

I confirm that I have read this submission and believe that I have an appropriate level of expertise to confirm that it is of an acceptable scientific standard.

# Reviewer report: Reviewer response for version 1

**DOI:** 10.5256/f1000research.166604.r299580

**Published:** 2024-08-23

**Authors:** Kevin S. Lang

**Affiliations:** 1 University of Minnesota, Saint Paul, MN, USA

This is an intriguing essay that urges for the reassessment of what is considered “life”. The author makes a case for refining definitions of “life” to aid in the pursuit of identifying lifeforms on planets other than Earth. They highlight existing frameworks, such as the selfish gene theory, in the context of replication of biological molecules, suggesting that lifeforms may have some fluidity beyond historical definitions. These ideas have significant value for shaping the perceptions of what life could look like beyond what is observed on Earth. In my opinion the essay could be improved significantly by the following considerations:

1. The author should consider providing a more explicit suggestion of what they think the definition should be tailored to. For example, is life strictly constrained by the replication of only organic molecules? Could replication of inorganic molecules be considered “life”? This is important as the author highlights that some organic molecules (ie genes) utilize organisms for their own replication. Parallel to this framework, things like limestone could be considered to replicate by utilizing marine life for its replication. Would this then be considered life?
2. In line with the above comment, would the new definition of life pertain to molecules that template their own replication or self-assembly? The author discusses the generation of biomolecules from inorganic substrates, citing the RNA theory of the origin of life. This is an example of self-assembly. Other examples of this beyond RNA and DNA exist on Earth, for example, prions and viral capsids. The distinction of these molecules is that they don’t necessarily involve templating their own replication. Rather, they drive their own self-assembly from simple substrates to more complex biomolecules. A clearer definition would enhance this essay from being a simple review of existing ideas to proposing a novel definition.
3. Lastly, the author highlights indexed essays that discuss the relationship between life and entropic forces. However, the way the author discusses the relationship between life and entropy is unclear. For example, the author states that DNA and RNA are in a state of high entropy. This is perplexing, given that these biomolecules are highly ordered, and thus fighting against entropy. Therefore, is the author stating that the new definition of life should be any molecules that are moving to a state of low entropy, and thus, driving themselves away from natural tendency of the 2 ^nd^ law of thermodynamics to increase entropy?

In summary, this is an intriguing essay that will benefit by a more explicit definition of what should be considered “life”.

Is the topic of the opinion article discussed accurately in the context of the current literature?

Partly

Are arguments sufficiently supported by evidence from the published literature?

Partly

Are all factual statements correct and adequately supported by citations?

Partly

Are the conclusions drawn balanced and justified on the basis of the presented arguments?

Partly

Reviewer Expertise:

DNA Replication, transcription, translation, microbiology, molecular biology, evolution.

I confirm that I have read this submission and believe that I have an appropriate level of expertise to confirm that it is of an acceptable scientific standard, however I have significant reservations, as outlined above.

# Response

**Published:** 2024-09-13

**Authors:** Zheng Liu

**Affiliations:** Guilin Medical University, China

Authors' point by point response to the Reviewer comments is as follows - 

 1.    I have considered this issue while writing my paper, but I have been very cautious. I am also concerned that my viewpoint may be too radical and difficult to accept. However, with the help of the F1000Research platform and the opportunity to answer questions, my response is: Yes. Traditional thinking defines life as having certain perceptions or consciousness, such as in humans, animals, and plants. However, the emergence of viruses has challenged this common understanding. A virus is merely a sequence of DNA or RNA, and although it is an organic substance (without consciousness, is it different from inorganic substances?), it constantly replicates and is also a form of life. I believe we can be bolder. In my article, I speculate that inorganic substances capable of self-replication can also be considered life, which is a novel viewpoint in this paper. Regarding the statement 'limestone could be considered to replicate by utilizing marine life,' I disagree because limestone is not self-replicating; its production results from the accumulation of products from marine biological activities. I think it is essential to emphasize self-replication, which involves actively replicating oneself.

 2.    This question is very interesting. The main point of the paper is that molecules capable of self-replication can be defined as life, similar to a piece of DNA (virus) that can self-replicate. If a molecule only drives its own self-assembly from simple substrates to more complex biomolecules, I believe this is growth, not replication, and therefore not life. I think the example of prions is also a form of life. Prion protein is coded by the PRNP gene. Prion proteins cannot self-replicate and cannot be considered life. However, while prions are composed of misfolded protein aggregates, they propagate by inducing conformational changes in normal prion proteins (PrP^C) to transform them into the abnormal, disease-causing form (PrP^Sc). The misfolded prion acts as a template, guiding the normal proteins to adopt the abnormal structure, exhibiting characteristics of life. This precisely supports the viewpoint of this paper that any self-replicating molecule, including misfolded prions, can serve as genetic material and ultimately become life. Additionally, I have included examples of prions in the revised version of my paper. Viral capsids are part of a virus and are composed of proteins encoded by viral genes. To my knowledge, I have not seen viral capsids self-replicate. If there were independent viral capsids that could self-replicate (no need for DNA or RNA), I would also define them as life. Thank you very much for your suggestion. I have added a clearer definition of life to my paper in the conclusion section and changed the title to " Life should be redefined: Any molecule with the ability to self-replicate should be considered life ". Additionally, it should be emphasized that self-replication should also include one molecule inducing another molecule to become itself (such as a misfolded prions induce prions). 

 3.    I believe there may be a misunderstanding. According to England's theory, DNA or RNA molecules exist in a high-energy (low entropy, ordered and organized) state, which makes them ordered. The replication of DNA or RNA molecules progresses towards a lower energy state, ultimately leading to a low-energy (higher entropy, disordered and disorganized). This process is driven by the second law of thermodynamics, which governs the behavior of all matter in the universe, including life. However, an increase in entropy driven by the second law of thermodynamics does not necessarily indicate life; only molecules with active self-replication functions are considered life. The second law of thermodynamics drives all matter in the universe towards disorder and heat death. For example, if a drop of ink is added to a cup of water, the ink will eventually distribute evenly among the water molecules due to the second law of thermodynamics, which is unrelated to life, vice versa. I am very grateful for your suggestions on my paper. I have been contemplating new perspectives on life during this period, and I have added new paragraph accordingly.

# Reviewer report: Reviewer response for version 1

**DOI:** 10.5256/f1000research.166604.r312177

**Published:** 2024-08-21

**Authors:** Denis Noble

**Affiliations:** 1 University of Oxford, Oxford, UK

This Review article proposes that there could be other self-replication molecules in addition to DNA & RNA. It also supports England's (2015) hypothesis that entropic forces are responsible for making matter adopt life-like physical properties.  It justifies this speculation using recent work by other authors. The review will be of most use to students searching for new leads on current thinking about the origin of life.

Is the topic of the opinion article discussed accurately in the context of the current literature?

Yes

Are arguments sufficiently supported by evidence from the published literature?

Yes

Are all factual statements correct and adequately supported by citations?

Yes

Are the conclusions drawn balanced and justified on the basis of the presented arguments?

Partly

Reviewer Expertise:

Cardiac electrophysiology, evolutionary biology.

I confirm that I have read this submission and believe that I have an appropriate level of expertise to confirm that it is of an acceptable scientific standard.

# Response

**Published:** 2024-09-13

**Authors:** Zheng Liu

**Affiliations:** Guilin Medical University, China

Thank you very much for recognizing the viewpoints presented in this paper. The open new mode of F1000Research allows me to express novel viewpoints and share them with other academic scholar. These viewpoints are often difficult to get accepted by traditional journals, but I believe that the emergence of any novel viewpoint will drive progress in science and thought.
